# Overlap of Primary Membranous Nephropathy, IgA Nephropathy, and Diabetic Nephropathy: A Case Report

**DOI:** 10.7759/cureus.49598

**Published:** 2023-11-28

**Authors:** Abdullah H Alghamdi

**Affiliations:** 1 Internal Medicine, Imam Mohammad Ibn Saud Islamic University, Riyadh, SAU

**Keywords:** renal disorders, coexistence, diabetic nephropathy, iga nephropathy, primary membranous nephropathy

## Abstract

The coexistence of primary membranous nephropathy (PMN), immunoglobulin A nephropathy (IgAN), and diabetic nephropathy (DN) in the same patient has been a subject of clinical and pathological investigation, yielding inconclusive results. The limited availability of cases and resource materials has hindered a comprehensive understanding of this phenomenon. We present the case of a 70-year-old Saudi Arabian man diagnosed with type 2 diabetes mellitus and nephrotic syndrome. A kidney biopsy revealed the coexistence of PMN, IgAN, and DN. The patient presented with an unusual and rare combination of PMN, IgAN, and DN. To address his condition, the patient consented to rituximab therapy and planned follow-up with the kidney transplant team. However, before the first dose of rituximab could be administered, the patient experienced severe septic shock secondary to pneumonia, which tragically led to his demise. The simultaneous occurrence of PMN, IgAN, and DN represents a rare and scarcely documented condition. The purpose of this article is to report this exceptional case, emphasizing the significance of further research to deepen the understanding of the underlying pathology behind these concurrent renal disorders. This report aims to shed light on the complexities of managing such complex cases and advancing therapeutic approaches in the future.

## Introduction

Membranous nephropathy (MN) is a kidney condition characterized by the accumulation of immune complexes in the basement membrane of glomeruli, leading to proteinuria, hypoalbuminemia, edema, and hyperlipidemia [1]. It can be divided into primary MN (PMN) with an unknown cause and secondary MN (SMN) caused by various conditions. PMN accounts for approximately 75-80% of MN cases and is typically caused by autoimmune factors [2]. Autoantibodies target podocyte surface antigens, such as PLA2R and THSD7A. These autoantibodies, such as aPLA2R-Ab, are valuable for diagnosis and assessing treatment response [3]. However, despite progress, the prognosis remains diverse, and personalized treatment remains challenging. Recent research has also shed light on other podocyte antigens' potential involvement in MN pathogenesis, indicating the need for further exploration.

Immunoglobulin A nephropathy (IgAN) is a common primary glomerulonephritis worldwide and a primary cause of renal failure. It has a diverse range of clinical presentations and affects patients of all ages and populations. Symptoms of IgAN vary from asymptomatic microscopic or macroscopic hematuria to more severe presentations such as nephritic syndrome, nephrotic syndrome, or rapidly progressive glomerulonephritis. It involves the production of an abnormal form of IgA subtype immunoglobulin, the formation of autoantibodies against this abnormal IgA, and the subsequent accumulation of immune complexes in the glomerulus, leading to kidney injury [4]. The diagnosis of IgAN is usually straightforward due to the dominant or co-dominant presence of mesangial IgA deposits [5]. The treatment and management of IgAN depend on the severity and the risk of progression to end-stage kidney disease (ESKD). There is still ongoing research to better understand the disease's pathogenesis and identify effective treatment strategies.

Diabetic nephropathy (DN) is a metabolic disorder and a leading cause of ESKD. Almost half of the patients with type 1 diabetes develop DN and reach ESKD. Furthermore, around 30% of individuals diagnosed with type 2 diabetes also experience DN, while 45% of patients undergoing dialysis have diabetes as their primary diagnosis, placing them in a vulnerable position with regard to cardiovascular disease [6]. DN is characterized by increased albuminuria, which indicates the progression of renal damage caused by defects in the glomerular filtration barrier. Potential therapies include the inhibition of SGLT2-mediated sodium-coupled glucose transport, and recently, finerenone, a novel, selective non-steroidal mineralocorticoid receptor antagonist, has shown renoprotective effects similar to inhibitors of the renin-angiotensin-aldosterone system [7]. Research has shed light on the mechanisms of progression of diabetic kidney diseases, including glomerular insulin signaling, oxidative stress, and endoplasmic reticulum stress. Understanding these mechanisms is crucial for developing effective treatments for DN and its complications.

Both IgAN and MN are commonly observed in patients with glomerulopathies. However, the occurrence of these two conditions together is rare, and the clinical implications of their coexistence in the same patient remain unclear. The situation becomes even more complex when DN is also diagnosed in conjunction with these conditions. Here, we report the case of a patient who was diagnosed with a combination of primary membranous glomerulonephritis, IgAN, and DN. This rare and unusual condition warrants documentation for further investigation to enhance our understanding of the underlying pathology of concurrent renal diseases.

## Case presentation

A 70-year-old Saudi male with a medical history of type 2 diabetes (diagnosed over 20 years previously), hypertension, and ischemic heart disease presented to the nephrology clinic with a two-week history of worsening bilateral lower limb edema. At presentation, his blood pressure was 164/109 mmHg. Laboratory tests revealed normocytic anemia, metabolic acidosis, and elevated creatinine. Urinalysis revealed +3 for protein and a high RBC count. Two years prior to presentation, his baseline labs showed serum creatinine of 1.27 mg/dL, with microalbumin:creatinine ratio of 24 mg/g creatinine and hemoglobin A1C (HbA1C) of 7% (A1C was 9.3% in 2018, 8% in 2018, 9.7% in 2011, 8.8% in 2010, 9.6% in 2009, and 8.3% in 2006 (first available A1C)).

**Figure 1:**
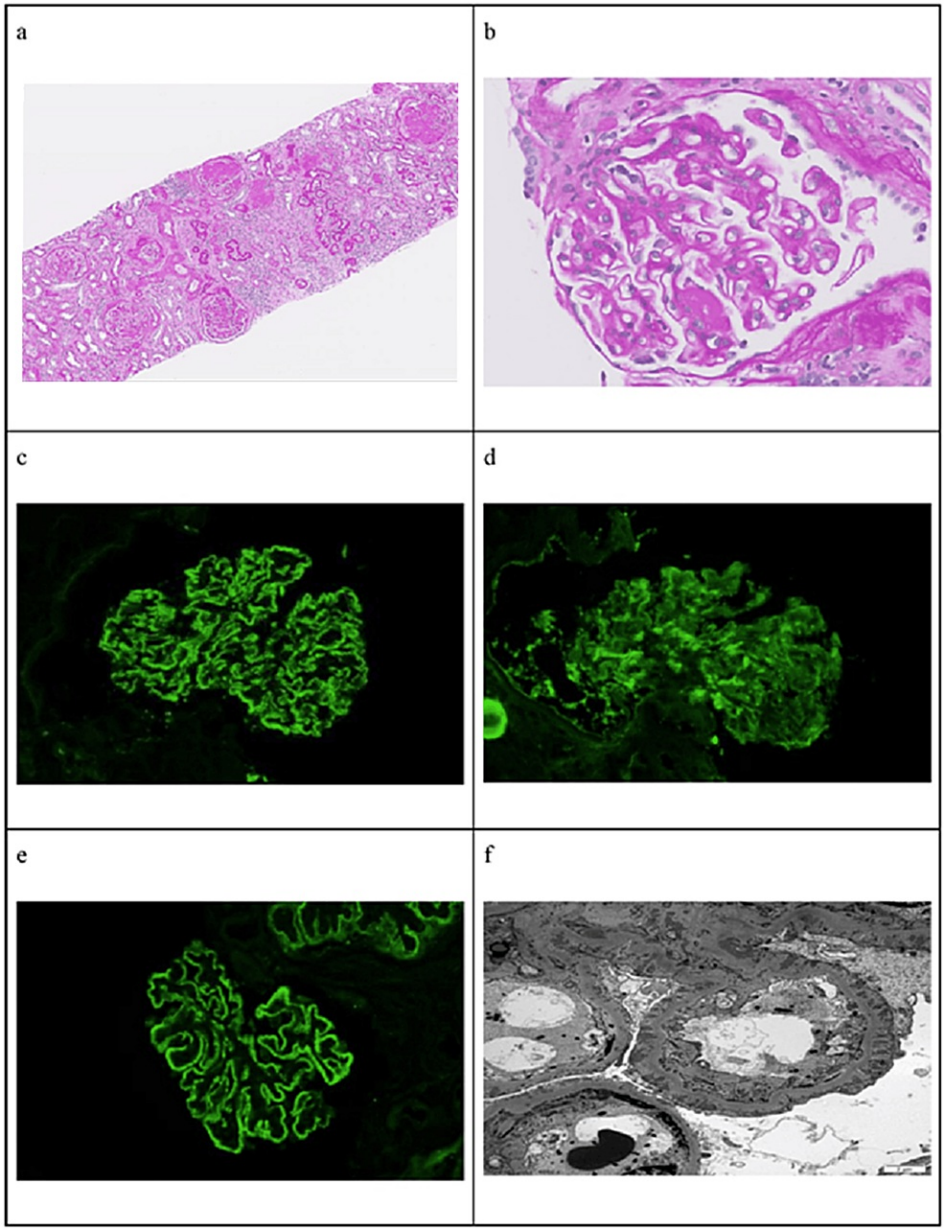
Renal biopsy findings suggestive of IgAN and MN. (a) Prominent interstitial fibrosis, globally and segmentally sclerotic glomeruli, and glomeruli with thickened capillaries, some with periglomerular fibrosis. (b) Light microscopy showing a glomerulus with thickened capillaries and mesangial expansion consistent with MN and IgA. (c) Immunofluorescence showing IgG in a granular pattern consistent with MN. (d) Immunofluorescence showing mesangial staining for IgA consistent with IgAN. (e) Positive anti-PLA2R stain in the glomerular capillary deposits. (f) Electron micrograph showing subepithelial spike and ball deposits consistent with MN. Glomerular capillary (subepithelial and intramembranous) and mesangial deposits. IgAN: immunoglobulin A nephropathy; MN: membranous nephropathy; PLA2R: phospholipase A2 receptor.

Further investigations showed that the patient's antinuclear antibodies (ANA) and complements were normal, but 24-hour urine protein was significantly elevated. Echocardiogram and kidney ultrasound were normal. Anti-phospholipase A2 receptor (anti-PLA2R) titer suggested a possible association with membranous glomerulonephritis. HIV, hepatitis B surface antigen, and hepatitis C antibodies were negative. A kidney biopsy was performed, and the histological examination showed overlapping glomerular stages II, III, and IV of primary membranous glomerulonephritis, along with evidence of IgAN and changes of DN with diffuse diabetic glomerulosclerosis (Figure 1). Additionally, severe interstitial fibrosis and tubular atrophy (75%) were observed on the biopsy. All test results have been summarized in Table 1 (January 2021).

**Table 1:** Reported and normal values of all test parameters. HbA1C: hemoglobin A1C; ANA: antinuclear antibody; anti-PLA2R: anti-phospholipase A2 receptor.

Parameter	Reported value	Normal value
Blood pressure (mmHg)	164/109	90/60–120/80
Hemoglobin (g/dL)	8.2	12-16
pH	7.22	7.35–7.45
HCO_3 _(mEq/L)	16	22–28
PCO_2_	40	35–45
Anion gap (mEq/L)	18	4–12
Creatinine (mg/dL)	6	0.7–1.3
Parathyroid hormone (pg/mL)	451	10–55
HbA1C	6% 2021	<5.7% normal
		5.7-6.4% pre-diabetes
		>6.4% diabetes
ANA	1:80 speckles appearance	<1:80
C3 (mg/dL)	106	82-185
C4 (mg/dL)	32.8	15-53
Anti-PLA2R	1:320	<1:10
Serum albumin g/dL	2.1	3.5-5
Urine analysis	+3 for protein High RBC count	Negative 4.7-6.1 cell/mcL
24-hour urine protein	14 g/day	150 mg/day

Considering the severity and complexity of the case, the patient was referred to a transplant center for transplantation assessment. Meanwhile, a multidisciplinary team held discussions regarding the best treatment option. After extensive discussion with the patient, rituximab was chosen as the treatment of choice to target the underlying autoimmune component associated with membranous glomerulonephritis.

However, before administering the first dose of rituximab, the patient developed severe septic shock secondary to pneumonia and was urgently transferred to a rural hospital. Despite aggressive management, the patient's condition rapidly deteriorated, and he passed away.

## Discussion

This case report describes a unique and challenging case of a 70-year-old Saudi male with a combination of PMN, IgAN, and DN. While previous reports have described combined PMN and IgAN cases, overlapping DN has not been observed. He et al. characterized patients with concomitant PMN and IgAN, highlighting higher levels of proteinuria, lower estimated glomerular filtration rate (eGFR), a higher likelihood of hypertension, and nephrotic syndrome in these individuals compared to those with IgAN alone. Additionally, patients with combined PMN and IgAN exhibited lower levels of urinary microscopic hematuria, gross hematuria, and plasma IgA [8].

The complex interplay between PMN, IgAN, and DN in both adults and children remains poorly understood. A potential role of genetics in the development of concurrent renal diseases has been suggested. Miyazaki et al. investigated the correlation between genetics and simultaneous MN and IgAN in two male siblings [9].

Histologically, PMN is characterized by extensive foot process effacement and subepithelial deposits, with granular IgG (often IgG4) deposits in the capillary wall under immunofluorescence [10]. Treatment outcomes for PMN vary, with some patients achieving remission while others require kidney transplantation. Spontaneous remission occurs in approximately 32% of cases within the first one to two years, but one-third of patients will progress to ESKD [11,12]. Attaining remission, as shown by Polanco et al., is associated with better long-term kidney survival [12].

In the case of IgAN, the disease exhibits diverse clinical manifestations ranging from mesangial hypercellularity to glomerular crescent formation. IgA deposits, along with IgG, IgM, and C3, are evident in the mesangium [13,14]. The prognosis varies widely, and up to 30% of patients may progress to ESKD [15]. Predictors of declining kidney function include the severity and persistence of proteinuria, hypertension, and low GFR [15,16]. Post-transplant, IgAN can recur in a significant percentage of patients, leading to graft dysfunction and loss [14].

Comparison of biopsy results between patients with concomitant PMN and IgAN and those with PMN only showed no significant differences in terms of proteinuria, hyperlipidemia rates, serum total cholesterol, serum lipoprotein cholesterol, hypertension rates, and gross hematuria rates [8]. Adding complexity to the case, the patient also had DN. Diabetes is a global health concern, affecting a substantial population and leading to severe complications if left unmanaged. Sulaiman reported that chronic tissue complications and organ damage can arise in diabetic individuals due to prolonged hyperglycemia [17]. Albuminuria is an early indicator of organ damage, specifically renal or glomerular diseases like DN. Within 10-20 years of diabetes onset, DN may develop in a significant proportion of patients, affecting approximately 20-40% of the diabetic population, ultimately leading to end-stage kidney disease.

The decision to use rituximab as the treatment modality was based on the elevated levels of anti-PLA2R, indicating a possible autoimmune component in primary membranous glomerulonephritis [18]. Rituximab, a monoclonal antibody that targets CD20-positive B cells, has shown promising results in the treatment of autoimmune glomerulonephritis [19]. However, the patient's untimely demise prevented the initiation of this therapy, highlighting the critical nature of managing coexisting medical conditions in patients with complex kidney diseases.

The coexistence of PMN, IgAN, and DN represents a complex and poorly understood phenomenon. Genetic factors are critical in the development of concurrent renal diseases. Treatment outcomes and prognoses for each condition vary widely, with some patients achieving remission while others progressing to ESKD. The unique case presented here sheds light on the potential interactions between these three renal disorders and underscores the importance of further research to unravel their intricate pathogenesis.

## Conclusions

The presence of multiple kidney pathologies and chronic medical conditions complicated the treatment approach. Despite efforts to initiate rituximab therapy for membranous glomerulonephritis, the patient's unfortunate demise due to severe septic shock emphasizes the importance of early recognition and management of complex kidney diseases in high-risk individuals. Further research and understanding of such rare combinations of glomerulonephritis are essential to improve patient outcomes in the future.

## References

[1] Gupta S, Pepper RJ, Ashman N, Walsh SB (2019). Nephrotic syndrome: oedema formation and its treatment with diuretics. Front Physiol.

[2] Ponticelli C, Glassock RJ (2014). Glomerular diseases: membranous nephropathy--a modern view. Clin J Am Soc Nephrol.

[3] van de Logt AE, Fresquet M, Wetzels JF, Brenchley P (2019). The anti-PLA2R antibody in membranous nephropathy: what we know and what remains a decade after its discovery. Kidney Int.

[4] Al Hussain T, Hussein MH, Al Mana H, Akhtar M (2017). Pathophysiology of IgA nephropathy. Adv Anat Pathol.

[5] Mahat A, Lageju N, Neupane D, Mishra U, Koirala S (2023). Immunoglobulin A nephropathy in remission: a case report. Ann Med Surg.

[6] Gnudi L, Gentile G, Ruggenenti P (2018). The patient with diabetes mellitus. Oxford Textbook of Clinical Nephrology.

[7] Rossing P, Burgess E, Agarwal R (2022). Finerenone in patients with chronic kidney disease and type 2 diabetes according to baseline HbA1c and insulin use: an analysis from the FIDELIO-DKD study. Diabetes Care.

[8] He JW, Cui DF, Zhou XJ (2022). Concurrent IgA nephropathy and membranous nephropathy, is it an overlap syndrome?. Front Immunol.

[9] Miyazaki K, Miyazaki M, Tsurutani H (2002). Development of IgA nephropathy 14 years after diagnosis of membranous nephropathy. Nephrol Dial Transplant.

[10] Fogo AB, Lusco MA, Najafian B, Alpers CE (2015). AJKD atlas of renal pathology: membranous nephropathy. Am J Kidney Dis.

[11] Dahan K, Debiec H, Plaisier E (2017). Rituximab for severe membranous nephropathy: a 6-month trial with extended follow-up. J Am Soc Nephrol.

[12] Polanco N, Gutiérrez E, Covarsí A (2010). Spontaneous remission of nephrotic syndrome in idiopathic membranous nephropathy. J Am Soc Nephrol.

[13] Khorsan R, Hanna RM, Ameen K (2019). Primary membranous nephropathy with concomitant IgA nephropathy. Saudi J Kidney Dis Transpl.

[14] Infante B, Rossini M, Leo S (2020). Recurrent glomerulonephritis after renal transplantation: the clinical problem. Int J Mol Sci.

[15] Le W, Liang S, Chen H (2014). Long-term outcome of IgA nephropathy patients with recurrent macroscopic hematuria. Am J Nephrol.

[16] Wyatt RJ, Julian BA (2013). IgA nephropathy. N Engl J Med.

[17] Sulaiman MK (2019). Diabetic nephropathy: recent advances in pathophysiology and challenges in dietary management. Diabetol Metab Syndr.

[18] Wang S, Deng Z, Wang Y, Bao W, Zhou S, Cui Z, Zheng D (2023). Monthly mini-dose rituximab for primary anti-PLA2R-positive membranous nephropathy: a personalized approach. BMC Nephrol.

[19] Chambers SA, Isenberg D (2005). Anti-B cell therapy (rituximab) in the treatment of autoimmune diseases. Lupus.

